# Generation of TALE nickase-mediated gene-targeted cows expressing human serum albumin in mammary glands

**DOI:** 10.1038/srep20657

**Published:** 2016-02-08

**Authors:** Yan Luo, Yongsheng Wang, Jun Liu, Chenchen Cui, Yongyan Wu, Hui Lan, Qi Chen, Xu Liu, Fusheng Quan, Zekun Guo, Yong Zhang

**Affiliations:** 1 College of Veterinary Medicine, Northwest A&F University, Yangling 712100, Shaanxi, China; 2 Key Laboratory of Animal Biotechnology of the Ministry of Agriculture, Northwest A&F University, Yangling 712100, Shaanxi, China

## Abstract

Targeting exogenous genes at milk protein loci *via* gene-targeting technology is an ideal strategy for producing large quantities of pharmaceutical proteins. Transcription- activator-like effector (TALE) nucleases (TALENs) are an efficient genome-editing tool. However, the off-target effects may lead to unintended gene mutations. In this study, we constructed TALENs and TALE nickases directed against exon 2 of the bovine β-lactoglobulin (BLG) locus. The nickases can induce a site-specific DNA single-strand break, without inducing double-strand break and nonhomologous end joining mediated gene mutation, and lower cell apoptosis rate than TALENs. After co-transfecting the bovine fetal fibroblasts with human serum albumin (HSA) gene-targeting vector and TALE nickase expression vectors, approximately 4.8% (40/835) of the cell clones contained HSA at BLG locus. Unexpectedly, one homozygous gene-targeted cell clone (1/835, 0.1%) was obtained by targeting both alleles of BLG in a single round of transfection. The recombinant protein mimicking the endogenous BLG was highly expressed and correctly folded in the mammary glands of the targeted cows, and the expression level of HSA was significantly increased in the homozygous targeted cows. Results suggested that the combination of TALE nickase-mediated gene targeting and somatic cell nuclear transfer is a feasible and safe approach in producing gene-targeted livestock.

The mammary gland bioreactor is a promising expression system for producing large quantities of exogenous proteins^1^,^2^. The integration of exogenous genes at the milk protein loci through gene-targeting technology is an ideal approach to avoiding position effects and in achieving high protein expression. TALENs are highly efficient and versatile tools for genome editing and have been successfully used in various organisms, including yeast^3^, plants^4^, viruses^5^, fishes^6^, insects^7^, and mammals^8^. However, double-strand breaks (DSBs) produced by TALENs are predominantly repaired by error-prone non-homologous end joining (NHEJ), which induces small insertions and deletions (indels) at the target sites^9^. Although the efficiency of TALEN-induced off-target is demonstrated to be lower than those of ZFNs and clustered regularly interspaced short palindromic repeat (CRISPR)/CRISPR associated (Cas) system, off-target events are also detected in TALENs-treated cells^10^. Minimizing the risk of unwanted gene mutation by employing certain strategies, such as introduction of DNA single-strand break (SSB) or nick, is also necessary for specific and precise gene modification. Previous studies demonstrated that zinc-finger nickases (ZFNickases) derived from a ZFN pair *via* D450 mutation of the FokI catalytic domain can induce SSBs, and nickase-induced SSBs are sufficient to stimulate homology-directed repair (HDR) without NHEJ in human and animal cells^9^,^11^,^12^. This finding suggested that nickase can improve the fidelity of gene modification. Therefore, TALE nickases exhibit a potential in mediating precise gene targeting at the mammary protein locus to produce mammary gland bioreactors.

Human serum albumin (HSA) is the most abundant plasma protein and is widely used in medical treatments and in biological pharmacy^13^. Therapeutic HSA is mainly derived from human plasma. However, the application of plasma-derived HSA (pdHSA) is limited by the availability of sources and the risk of infection by pathogenic microorganisms^14^. Thus, recombinant HSA (rHSA) has become an ideal pdHSA candidate. β-Lactoglobulin (BLG) is a major milk protein in the whey of cow, accounting for up to 12% of the total milk protein and it is also a major allergen^15^. Hence, BLG is a suitable locus for gene targeting to produce mammary gland bioreactors. This study tested the feasibility of targeting HSA gene in exon 2 of the bovine BLG locus by TALE nickase and determined the efficiency of HSA expression in the milk of gene-targeted cows generated by somatic cell nuclear transfer (SCNT).

## Results

### Activity of TALENs

**Figure 1:**
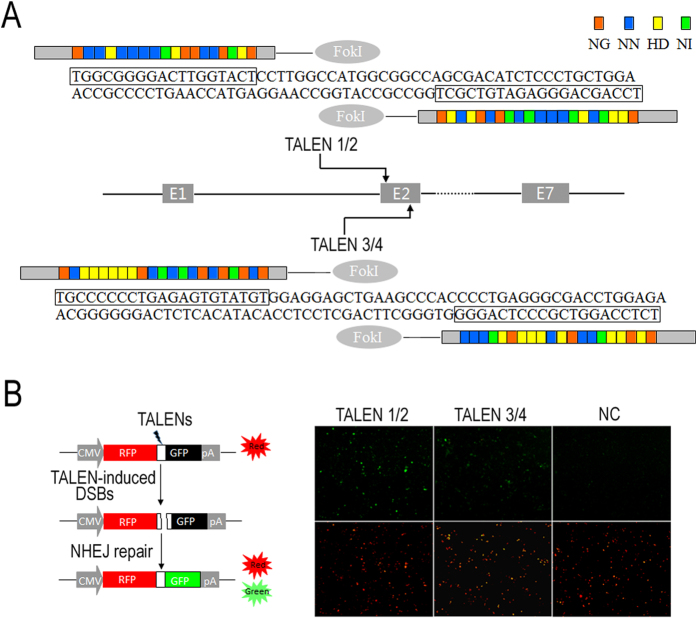
Detection of TALEN activity. (**A**) Location and sequences of two pairs of TALEN against bovine BLG. (**B**) Rapid detection of NHEJ-inducing activity using RFP–GFP reporter system in HK293 cells. (Left) Schematic of illustration for RFP–GFP reporter system assay; (right) fluorescence images of the cells at 36 h post-transfection. NC, cells transfected only with the reporter vector used as negative control. All images were obtained at 100× magnification.

We designed two pairs of TALENs directed against exon 2 of the bovine BLG locus (Fig. 1A). To estimate the activity of the TALEN pairs, we used the RFP–GFP reporter system (Supplementary methods and materials) to rapidly test their cleavage activity in HK293 cells (Fig. 1B). The results showed that both pairs of TALEN1/2 and TALEN3/4 restore GFP expression, indicating that TALENs can cleave the target BLG sequence, can trigger the NHEJ repair pathway, and can induce gene mutation. The expression level of GFP was higher in TALEN1/2 than in TALEN3/4 (Fig. 1B).

### Construction and evaluation of TALE nickases

**Figure 2:**
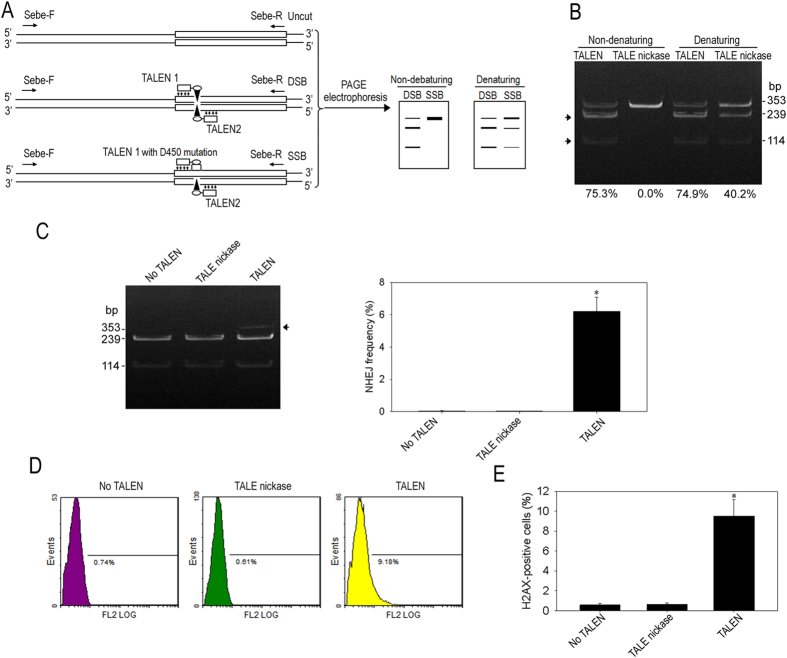
Detection of strand-specific nicking activity of TALE nickase. (**A**) Illustration of the expected digestion patterns of DNA products following *in vitro* cleavage with TALENs and TALE nickase. (**B**) *In vitro* assessment of cleavage activity of TALENs and TALE nickase under non-denaturing and denaturing conditions. The percentages of the cleaved products (indicated by black arrows) are shown at the bottom of each lane. (**C**) Detection of gene mutation in BFFs treated with TALENs and TALE nickase using surveyor nuclease assay by *NcoI*. (Left) *NcoI* digestion of PCR products analyzed using PAGE. Arrows indicate the uncleaved products. (Right) Statistical results of NHEJ efficiency in the cells treated with nuclease and nickase based on the result of *NcoI* digestion of PCR products. (**D,E**) Flow cytometric analysis of cellular γH2AX expression. (**D**) Percentage of γH2AX-positive cells analyzed using flow cytometry. After flow cytometric analysis of cellular γH2AX expression levels, a gate was drawn (black line in the figures) to encompass about 1% of BFFs of negative control. (**E**) Statistical results of cellular γH2AX expression. The columns indicate the fraction of γH2AX-positive cells of the transfected cells. Columns in (**C**,**E**) represent the average of three experiments. *Statistical significance increases compared with the no TALEN sample (*P* < 0.05).

We selected TALEN1/2, which exhibited high cleavage activity, in producing TALE nickases. The amino acid D450 of the FokI catalytic domain in the left TALEN was mutated. The amino acid residue number refers to the position of mutation in the full-length FokI endonuclease. *In vitro* cleavage activity detection showed that >75% of PCR products containing the target site were cleaved into two small fragments by TALEN1/2 under non-denaturing and denaturing conditions (Fig. 2A,B). However, the cleavage products cannot be separated into two small fragments by the corresponding TALE nickase under non-denaturing condition (Fig. 2B). When the cleavage products were resolved by PAGE under denaturing condition, 40.2% of the products were cleaved, and a full-length linear fragment remained observable (Fig. 2B). This result indicated the D450 mutation of the FokI catalytic domain in TALEN1 can create a TALE nickase with strand-specific nicking activity.

The sequence of spacer between the binding sites contains an *NcoI* site. The TALENs-induced NHEJ at the target site might prevent the cutting of *NcoI.* This phenomenon reflects the frequency of the TALEN- or TALE nickase-mediated cleavage of the endogenous target locus that was imperfectly repaired by NHEJ. Surveyor nuclease assay by *NcoI* revealed that the frequency of TALENs-induced NHEJ was 6.2% ± 0.8% (*n* = 3), whereas there was no obvious NHEJ existed in TALE nickase-treated cells and in the control sample (Fig. 2C). This finding showed that no gene mutation occurred in the bovine fetal fibroblasts (BFFs) transfected with TALE nickase, demonstrating that nickase-induced SSBs did not mediate the NHEJ DNA repair pathway. Overall, these data demonstrated that D450 mutation in one of the two TALENs generated a strand-specific TALE nickase without inducing NHEJ.

To further test the strand-specific nicking activity of TALE nickase, a genome-wide evaluation of DSB formation was evaluated using γH2AX staining, a key component of DSB-induced repair foci. The results of flow cytometric analysis results showed that the amount of γH2AX^+^ cells was significantly higher in the TALEN1/2 group (9.5% ± 1.7%, *n* = 3) than in the TALE nickase-treated cells (0.6% ± 0.2%, *n* = 3, *P* < 0.05) (Fig. 2D,E). No obvious difference existed in percentage of γH2AX^+^ cells between the TALE nickase-treated cells and no TALEN control cells (Fig. 2D,E). This observation indicated that TALE nickase did not cause genome-wide DSB, thereby preventing the potential occurrence of unintended gene mutations. This result was consistent with that of the surveyor nuclease assay.

### Gene addition efficiency and cell apoptosis in BFFs

**Figure 3:**
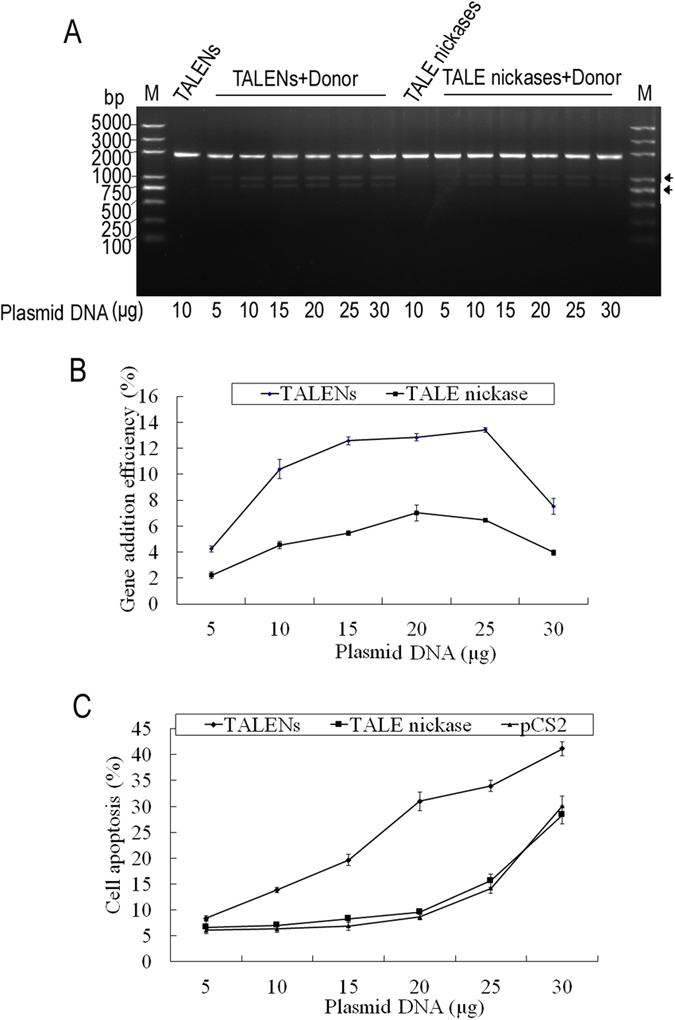
Comparison of gene addition efficiency and cell apoptosis mediated by TALENs and TALE nickases. (**A**) Result of gene addition efficiency detected by RFLP assay. Arrows indicate the cleaved products. (**B**) Statistical results of gene addition efficiency mediated by different doses of TALENs and TALE nickase. (**C**) Statistical analysis of cell apoptosis in cells transfected by different doses of TALENs and TALE nickase.

To compare the gene addition efficiency and cell toxicity of TALENs and TALE nickases, the BFFs were transfected with increasing amounts of expression vectors ranging from 5 μg to 30 μg in 1 × 10^6 ^cells and 400 μL electroporation buffer. The gene addition efficiency was evaluated by restriction fragment length polymorphism (RFLP) assay, which revealed a dose-dependent gene addition efficiency of TALENs or TALE nickases in the endogenous BLG locus. The optimal gene addition efficiencies were 13.4% and 7.0% for TALENs and TALE nickases at the amounts of 25 and 20 μg, respectively (Fig. 3A,B). Moreover, the gene addition efficiency of TALENs was higher than that of TALE nickases (*P* < 0.05). By contrast, the RFLP could not be detected in cells treated without donor plasmid (Fig. 3A). The frequency of apoptotic cells also increased with increasing doses of TALENs or TALE nickases, and a significant increase was observed at the amounts of 20 and 25 μg, respectively (Fig. 3C). The cell apoptosis assay also revealed that the cell toxicity of TALENs was higher than that of TALE nickases (*P* < 0.05). In addition, no difference existed between TALE nickases and pCS2 (no TALEN control). Our data demonstrate that the TALE nickase-mediated gene addition efficiency was reduced in compared with that of TALENs, but the former exhibited reduced toxicity.

### HSA gene targeting at BLG locus in bovine genome by using TALE nickase

**Figure 4:**
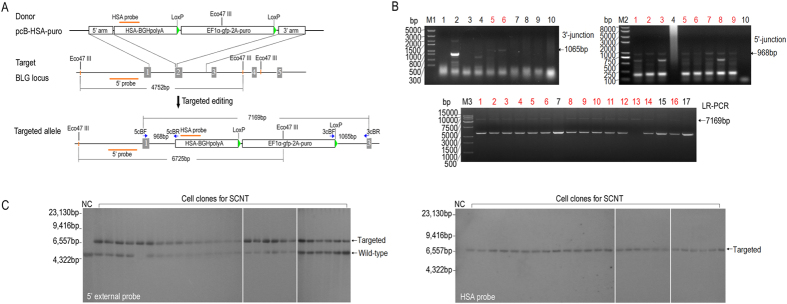
HSA gene targeting at BLG locus by TALE nickase-mediated HDR in BFFs. (**A**) Schematic of the targeting strategy for BLG locus. Southern blot probes and primers for PCR detection are shown as red lines and blue arrows, respectively. Gray boxes indicate the exons of BLG. HSA-BGHpolyA, HSA encoding sequence followed by BGHpolyA; and EF1α-gfp-2A-puro, elongation factor 1α promoter followed by GFP gene, a 2A self-cleaving peptide sequence and puromycin resistance gene. (**B**) PCR detection of drug-resistant cell clones. (Top) Left image shows the results of the 3′ junction PCR performed on drug-resistant cell clones using the primers 3cBF/R, and the right image shows the results of the 5′ junction PCR performed on 3′ junction- positive cell clones using primers 5cBF/R. (Bottom) Results of the LR-PCR performed on the 5′ junction positive cell clones using the primers 5cBF/3cBR. Lane 13 shows the bi-allelic targeted cell clone; the expected PCR products are shown by arrows. The red numbers indicate the PCR-positive clones. (**C**) Results of Southern blot conducted on cell clones with normal chromosome. 5′-probe (left) detected a 6.73 kb targeted fragment and 4.75 kb wild type fragment; HSA probe (right) detects a 6.73 kb targeted fragment.

We constructed the HSA gene-targeting vector pcB-HSA-puro (Supplementary Fig. S1) to test the efficiency of TALE nickase-mediated homologous recombination. The supercoiled pcB-HSA-puro with TALE nickase expression vectors were co-transfected into three BFF cell lines obtained from different female Holstein–Friesian dairy fetuses (Supplementary methods and materials). Up to 835 stably transfected cell clones were obtained through selection by puromycin (Table 1). To confirm the occurrence of gene insertion at the target site, approximately 75% of the cells of each clone were seeded in 48-well plates for extended culture; the rest were transferred into a PCR reaction tube and then treated as described previously^16^. Junction PCR and long-region (LR) PCR were performed on 2 μL of the cell lysates, and expected bands were observed in the samples of some clones (Fig. 4A,B). Approximately 7.3% (61/835) of the clones were 3′-junction positive, and 78.7% (48/61) of them were detectable in 5′-junction PCR assessment; 4.8% (40/835) of the cell clones were LR-PCR-positive (Table 1). Unexpectedly, one gene-targeted cell clone (TH1020-23) was identified as homozygous (1/835, 0.1%) (Fig. 4B, LR-PCR lane 13). The karyotypes of the non-senescent cell clones were tested, and all clones exhibited normal chromosome numbers. Prior to nuclear transfer, the cell clones with normal chromosomes were detected using Southern blot (Supplementary methods and materials) to further exclude the false positive clones. Using the 5′ external probe, we detected two bands (4.75 and 6.73 kb) in the heterozygous clones and one band (6.73 kb) in the homozygous clone (Fig. 4C, left). The upper band characterized the gene insertion, whereas the lower band represented the endogenous BLG allele. The HSA probe results also showed that a 6.73 kb strand was detectable in all of the PCR positive clones (Fig. 4C), indicating an approximately 4.8% efficiency of targeting knock-in of HSA into the TALE nickase-mediated BLG locus of BFFs.

### Generation of HSA gene-targeted cows via SCNT

Table 2 shows that three gene-targeted cell clones derived from each cell line were selected for SCNT. The cell clone TH1020-23 is homozygous. The blastocysts rate was approximately 20%, and a total of 377 blastocysts were transferred into the uterine horn of 126 recipients. Ninety days after embryo transfer, 28 recipients (22.2%) were pregnant. Finally, 10 out of the 28 pregnancies developed to term and produced living cloned calves. However, four cloned calves died several days after birth, and the six remaining calves lived healthily. Moreover, two out of the six cloned calves were produced from the homozygous cell clone TH1020-23.

**Figure 5:**
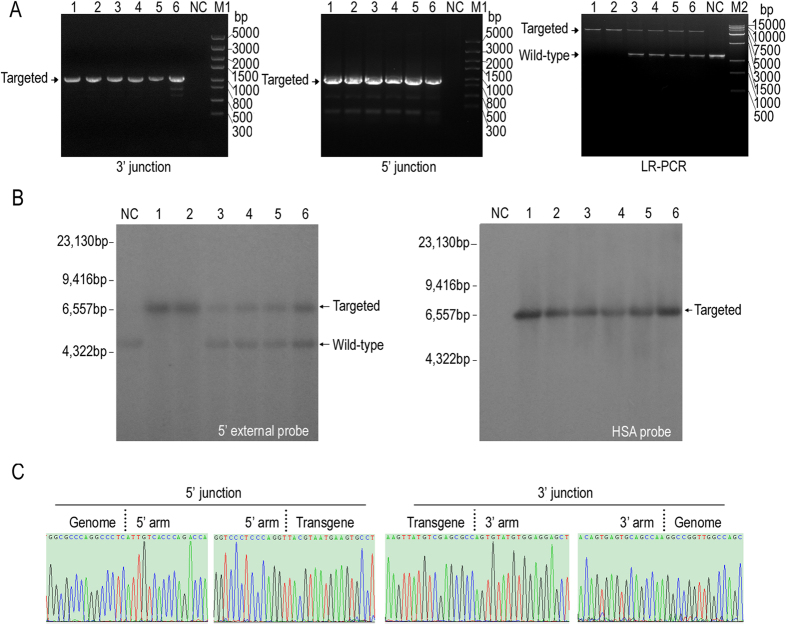
Detection of HSA gene-targeted cows. (**A**) Results of junction PCR detection and LR-PCR performed on six gene-targeted cows that survived for more than one month. (**B**) Results of Southern blot performed on the six targeted cows. The expected PCR products are indicated by arrow. (**C**) Sequence analysis of the junctions between the transgene and genome DNA corresponding to homologous recombination events. 1 and 2, homozygous targeted calves; 3–6, the heterozygous targeted calves; NC, the genomic DNA isolated from non-transgenic cow used as negative control.

To confirm whether the cloned calves were gene-targeted cows, the genomic DNA isolated from their blood of cloned calves was used as template for PCR detection and Southern blot analysis. Positive results for junction PCR and Southern blot were obtained from the six calves (Fig. 5A,B). The junction PCR products were subcloned into pMD18-T and then sequenced to further confirm the precise addition of HSA gene at the BLG locus. The results showed that the HSA-EGFP-puro cassette was successfully inserted into the target without gene mutation on either side of the two homologous arms (Fig. 5C). In addition, the PCR and sequencing detection of the off-targets using the primers around the nine potential off-target loci revealed no off-target events in the cows (Supplementary Table S2). This finding indicated the feasibility of producing gene-targeted cows with precise gene addition of long fragments at the BLG locus by combining TALE nickase with SCNT.

### Efficient HSA expression in gene-targeted cow’s milk with reduced BLG

**Figure 6:**
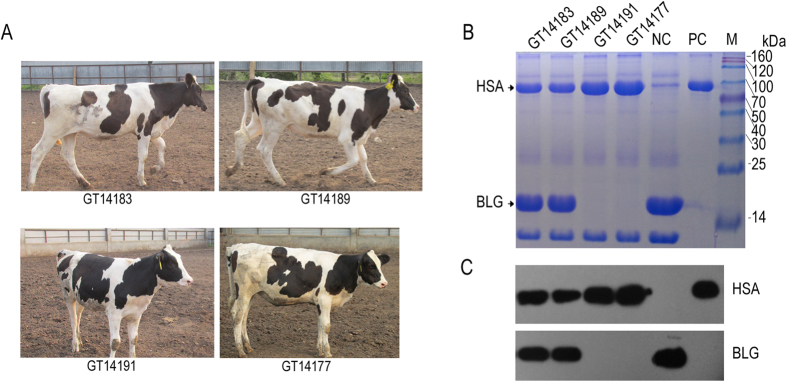
rHSA analysis and BLG expression in the targeted cows’ milk. (**A**) The four targeted cows used in hormone-induced lactation. The photographs of the four cows were taken by the first author Yan Luo. (**B**) Analysis of HSA expression in milk using SDS-PAGE. (**C**) Analysis of HSA and BLG expression levels in milk using Western blot. NC, milk from non-transgenic cow used as negative control; PC, pdHSA (Sigma) used as positive control; GT14183 and GT14189 are heterozygous targeted calves; GT14191 and GT14177 are homozygous targeted calves.

Four 5–7 months old prepuberal gene-targeted calves (heterozygous GT14183 and GT14189 from the BFF11-21 and BFF07-23 cell lines, respectively, homozygous GT14191 and GT14177 from the BFF10-20 cell line) (Fig. 6A) weighing 165 to 191 kg were hormonally induced into lactation by 7 daily injections of estrogen and progesterone as previously described^17^. Milk samples were sterilely collected by hand milking, and 1 ml milk of each sample was defatted by centrifugation. Casein proteins were removed from the skimmed milk *via* acid precipitation. Finally, we obtained 0.7–0.8 ml whey from each sample, and the resultant samples were diluted in PBS at 1:5 ratio. Each sample (10 μL) was then used for SDS-PAGE and Western blot analysis. A strong band of approximately 67 kDa was observable in the gene-targeted cows’ milk compared with the milk sample from the non-transgenic cows (Fig. 6B). Moreover, Western blot analysis showed an obvious band in the gene-targeted cows’ milk, and the band size was exactly the same as that of the native HSA standard (positive control). By contrast, no signal was detected in the non-transgenic cows’ milk (negative control) (Fig. 6C). The expression levels of BLG in the heterozygous targeted calves were also reduced, whereas no BLG expression was detected in the milk of the homozygous targeted calves (Fig. 6C). In addition, quantification of HSA expression by ELISA showed that the HSA expression levels in the milk of the targeted calves GT14183, GT14189, GT14191 and GT14177 were approximately 2.31, 2.15, 3.39 and 3.51 g/L, respectively. This result confirmed that the regulatory sequences of endogenous BLG can efficiently direct exogenous HSA gene expression in the mammary glands of the gene-targeted cows.

### Characterization of rHSA

**Figure 7:**
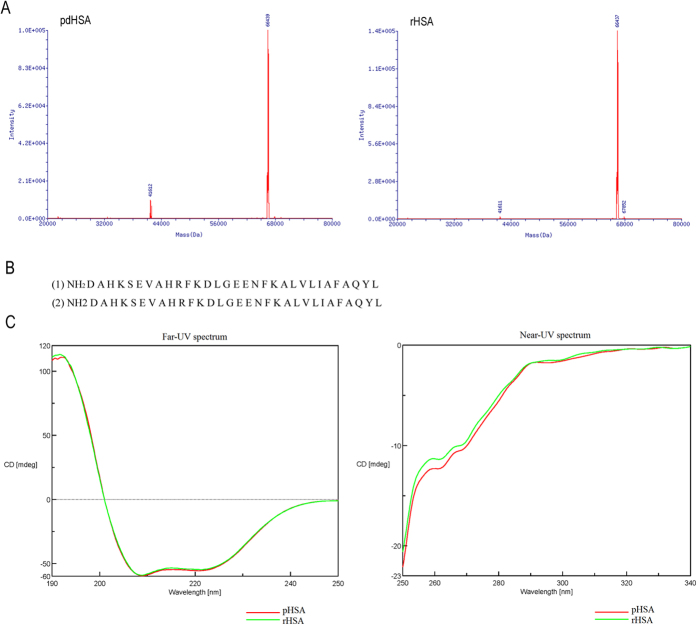
Characterization of rHSA. (**A**) Molecular mass of pdHSA (left) and rHSA (right) analyzed using Triple-TOF 5600^+^. (**B**) N-terminal 31 residues of pdHSA (1) and rHSA (2) tested using Edman degradation. (**C**) Structural analysis of rHSA. Secondary (left) and tertiary(right) structure of rHSA (green) and pdHSA (red) as determined by CD spectra in far- and near-UV regions.

Molecular-mass determination, N-terminal sequencing, and CD spectroscopy assays (Supplementary methods and materials) were conducted to characterize the molecular weight, primary sequence, and advanced structure of rHSA, respectively, for comparison with those of the pdHSA (Sigma), which is used as a standard. The results showed that the molecular mass and N-terminal in 31 residues of expressed rHSA were identical to those of pdHSA (Fig. 7A,B). Consistent with those of previous reports, the rsults of the far-UV CD spectroscopy showed a spectrum of mostly helical proteins for rHSA and pdHSA, and the two spectra were exactly similar to each other (Fig. 7C). Moreover, the near-UV CD spectra of rHSA matched that of pdHSA (Fig. 7C), indicating that rHSA was correctly folded in bovine mammary glands. This finding also implied that the activity of rHSA is similar to that of the native HSA, thereby further detecting of rHSA activity.

## Discussion

In this study, we constructed a TALEN nickase against exon 2 of the BLG locus for efficient and safe genome editing in bovine. By using TALE nickase combined with SCNT, we obtained two homozygous and four heterozygous targeted cows that expressed high level of recombinant HSA in their mammary glands. BLG was absent in the milk of the homozygous targeted cows, whereas the BLG in the milk of heterozygous targeted cows was reduced. Moreover, the recombinant protein was correctly folded and exhibited highly similar characteristics highly similar to those of the native protein.

The recent emergence of ZFNs, TALENs, and CRISPR-Cas system has largely promoted the development of gene-targeting technology^18^,^19^. These programmable nucleases have been applied in various plant, animal, and human cells^20^,^21^,^22^. However, these nucleases exert off-target effects on treated cells^23^,^24^. NHEJ-mediated indels at on- and off-targets can lead to unintended gene mutation. Therefore, minimization of off-target effects is important for accurate and safe genome editing. Researchers demonstrated that the D450 mutation of FokI in one of the two programmable nucleases can create a strand-specific nickase^9^,^11^. Wu *et al.*^25^ reported that TALE nickase mediates highly efficient targeted transgene integration at the human multi-copy ribosomal DNA locus. We also previously demonstrated that nickases can induce HDR but not NHEJ for gene targeting at the bovine β-casein locus and at the intergenic region between surfactant protein A1 (SFTPA1) and methionine adenosyltransferase I alpha (MAT1A) (the M-S locus) in bovine genome^12^,^26^. In the present study, we constructed a TALE nickase against bovine BLG locus and tested the strand-specific nicking activity of nickases in BFFs. Consistent with the previous reports, we found that nickases induced SSB and restricted DNA repair to HDR at the target. No increase in genome-wide DSB formation was detected in nickase-treated cells compared with the control group (Fig. 2). Similarly, Wang^11^ and Kim^9^ reported that CCR5-specific ZFNickases efficiently restrict DNA repair to HDR without significant levels of undefined indels, minimize cytotoxicity, and promote precise of nuclease-induced genome engineering in human cells. These studies demonstrated that ZFNickases and TALE nickases are safer than ZFNs and TALENs. Moreover, nickases can largely improve the reliability of gene editing and is useful in mediating precise gene modification *via* HDR in various loci.

TALENs are powerful and rapid gene-editing tools and thus are widely used in various species. The knockout efficiency of TALENs in mammalian cells is up to 54%^27^,^28^. However, the knock-in efficiency is not very high. TALENs stimulate homologous recombination of full-length β-globin cDNA to the endogenous locus in 19% of transfected K562 cells^28^. In addition, the efficiency of the TALENs-mediated homologous recombination at the BLG locus in goat fibroblast cells is 13.6%^16^. The current study, we tested the feasibility of TALE nickase-mediated gene addition in BFFs. We obtained the optimal gene addition efficiency (7.0%) with low cytotoxicity (9.6%) when the amount of nuclease expression vectors was increased up to 20 μg. Moreover, the TALE nickase-mediated gene addition efficiency was reduced in compared with that of TALENs-mediated one (7.0% vs. 12.8%), although the former shows reduced toxicity (9.6% vs. 31.0%). Our findings reported here are consistent with the previous results^25^.

The repair mechanism stimulated by SSB is different from that induced by DSB, resulting in low nickase- mediated targeting efficiency^11^. AAVS1-specific ZFNickase showed a 20-fold reduction in insertions compared with the corresponding ZFNs in K562 cells^11^. The homologous recombination efficiency decreased from 25% in the K562 cells treated with CCR5-specific nuclease to 9% in the cells treated with CCR5-specific nickase^9^. Liu *et al.*^12^ found that the integration efficiency mediated by β-casein-specific ZFNs was 15.7% in BFFs, and the efficiency was reduced to 3.8% in nickase-treated cells. These studies demonstrated that the knock-in efficiency of the nickase was reduced compared with the corresponding nuclease. However, the knock-in efficiency was sufficient for SCNT to produce gene-targeted animals. Moreover, we obtained one homozygous gene-targeted cell clones in a single round of targeting. Although the frequency was very low, our result demonstrated the feasibility of targeting in both alleles of endogenous gene locus by TALE nickase.

Many research groups have studied rHSA production in animal milk. The milk of transgenic mice yields various rHSA expression levels, with the highest level reaching 20 g/L^29^,^30^,^31^. In addition, the rHSA expression level in milk of transgenic cows varied from 1–2 g/L to 40 g/L^32^. However, the low milk yield of transgenic mice cannot meet the market demand, whereas the lactation duration of transgenic cows exhibiting the highest rHSA expression level is significantly short and cannot be used for further study. The inability of random addition of genes in controlling the gene insertion site and the gene copy number may lead to several unwanted results. Researchers found that the copy number of transgene in cloned cows exhibiting high rHSA expressing and abnormal lactation duration was approximately 250, whereas that of the cloned cows exhibiting low expression and normal lactation duration was 1–5^32^. Researchers suggested that milk thickness is ascribed to the obvious shortening of lactation duration^32^. Gene targeting is an ideal approach in avoiding the problems caused by random addition. To our knowledge, no HSA gene-targeted cows have so far been reported. Moghaddassi *et al.*^33^ demonstrated the TALEN-mediated replacement of BSA for HSA and the integration of an HSA transgene for the production of recombinant HSA in cows’ milk, and they obtained transgenic bovine embryos from the targeted fibroblasts through SCNT. Their work laid the foundation for the future generation of transgenic rHSA cattle. In the present study, we selected BLG as our target and we attempted to add HSA gene via TALE nickase-mediated homologous recombination to produce HSA expression mimicking the endogenous BLG. We detected the rHSA expression level and the initial characteristics of the protein. The HSA expression level in the milk of heterozygous targeted calves was approximately 2.5 g/L, and that in the milk of homozygous targeted calves was higher by up to 3.5 g/L. Moreover, the molecular mass, primary structure, and advanced structure were highly similar to those of the natural HSA (Fig. 7). This finding indicated that the rHSA expression targeted at this predetermined site properly mimicked the endogenous bovine BLG and can be correctly folded in the bovine mammary glands. Furthermore, the gene copy number was single in heterozygotes and two in homozygotes, and the off-target events and gene mutations were undetectable. Our results suggest that TALE nickases mediated accurate gene editing, and gene insertion into the target site used in our study enables efficient exogenous gene expression in gene-targeted cows.

In conclusion, TALE nickase can efficiently target HSA gene into the BLG locus with nearly no off-target effects on bovine genome. This behavior facilitated high expression of recombinant protein in the milk of gene-targeted cows. In addition, TALE nickase combined with SCNT can promote wide application of gene-targeted cows in agriculture and biomedicine.

## Materials and Methods

### Animals

All animal procedures and study design were conducted in accordance with the Guide for the Care and Use of Laboratory Animals (Ministry of Science and Technology of China, 2006). All experiments were approved by the Care and Use of Animals Center of the Northwest A&F University. The bovine ovaries were collected from Tumen abattoir, a local slaughterhouse in Xi’An, P. R. China. The fetuses and recipient cows were obtained from Yangling Keyuan Cloning Co., Ltd. All efforts were made to minimize animal pain, suffering, and distress.

### Construction of TALEN expression vector and nickase

The online software TAL Effector-Nucleotide Targeter designed the TALENs targeting the exon 2 of BLG (GenBank: X14710.1), which was selected in accordance with the previously described guidelines^34^. TALEs was constructed based on single-unit vectors (Kangwei, China) recognizing the individual nucleotides A, T, C, and G using the “unit assembly” strategy^35^. Finally, the assembled TALEs were inserted into the vectors pCS2-FokI-PEAS and pCS2-FokI-PERR (Kangwei, China) containing FokI cleavage domain to construct customized TALENs. Nickase generation was conducted as described previously^11^. In a typical procedure, a FokI domain D450A mutation was introduced into TALEN1 encoding plasmid by site-directed mutagenesis using the QuikChange Site-Directed Mutagenesis Kit (Stratagene) according to the manufacturer’s instructions.

### *In vitro* DNA cleavage assessment

The primers Sebe-F and Sebe-R were used in amplifying a target-site containing 353-bp fragment from the cattle genomic DNA (Supplementary Table S1). The PCR product was purified by AxyPrep kit (Axygen, USA). The TALENs and TALE nickases were synthesized *in vitro* using TnT® SP6 Quick Coupled Transcription/Translation System (Promega, Madison, WI, USA) according to the manufacturer’s instructions. An appropriate amount of the purified PCR product was mixed with TALENs or TALE nickases in CutSmart^TM^ buffer (NEB) and then digested at 37 °C for 2 h. Both denatured and non-denatured digested products were run on a 10% PAGE, stained with DuRed nucleic acid, imaged with E-Gel imager, and then quantified using ImageJ software (NIH, Frederick, MD). The cleavage activity was calculated by measuring the ratio of the total cleaved densitometry to total densitometry.

### *In vivo* DNA cleavage assessment by surveyor nuclease assay

The BFFs were seeded in 60 mm dishes at a density of 1 × 10^6^ and then cultured for 24 h. The cells were then transfected with 3.2 μg plasmids encoding TALENs or TALE nickases by FuGENE® HP (Roche, Mannheim, Germany). After 48 h of transfection, the genomic DNA of the cells was isolated using genomic DNA extraction kit (Tiangen, Beijing, China). Subsequently, the regions around the target were amplified from the genomic DNA by PCR using the primers Sebe-F and Sebe-R (Supplementary Table S1). The purified PCR products were digested by *NcoI* (NEB) to test the modification efficiency. The digested products were run on a 10% PAGE, stained with DuRed nucleic acid, and then imaged with E-Gel Imager. The NHEJ frequency was quantified by measuring the ratio of the uncleaved densitometry to total densitometry using ImageJ software. This experiment was repeated thrice.

### Assessment of genome-wide DSB formation by γH2AFX staining

The genome-wide DSB formation was assessed using γH2AX staining. The analysis of γH2AX staining was performed as previously described^36^. In a typical procedure, the BFFs were transfected with 0.4 μg of each of the nuclease/nickase expression plasmids using FuGENE® HP (Roche, Mannheim, Germany). The cells transfected with 0.8 μg pCS2, the skeleton plasmid for the TALEN expression plasmids pCS2-FokI-PEAS and pCS2-FokI-PERR, were used as control group (no TALEN). The cells were collected 2 days post-transfection, fixed in 75% ethanol, permeabilized with 0.2% saponin in blocking buffer (PBS, 1% BSA), incubated with anti-γH2AX monoclonal antibody (Upstate, 1:1,000) for 2 h at 4 °C, and then incubated with phycoerythrin (PE)-conjugated goat anti-mouse immunoglobulin G antibody (1:500, Santa Cruz) for 30 min at 4 °C. The BFFs treated only with the PE-conjugated secondary antibody were used as negative control. The percentage of γH2AX^+^ cells was determined using flow cytometry (Beckman Coulter, Krefeld, Germany).

### Profiling gene addition efficiency and cell apoptosis

Gene addition efficiency and apoptosis were examined in BFFs at different concentrations of TALENs or TALE nickase. The frequency of gene modification by homologous recombination was evaluated by a RFLP assay as described previously^12^. In a typical procedure, a donor DNA plasmid p BLG-EGFP was constructed to introduce a unique Hind III site at the cleavage sequence. In this construct, a 1571-bp homology arm, which was amplified from bovine genomic DNA by the primers DarmF/R (Table S1), was interrupted with a 6-bp sequence engineered to introduce a Hind III recognition site at the position of the cleavage sequence using QuikChange Site-Directed Mutagenesis Kit (Stratagene). We introduced this donor plasmid along with different amounts of TALENs or TALEN/TALE nickase plasmids (5–30 μg) into the BFFs. The primer pair DinF/R (Supplementary Table S1) located outside the region of homology arm was used in the PCR-amplification of a 1829-bp fragment from the genomic DNA extracted 48 h post-transfection. The PCR products were digested with Hind III. The products were resolved on a 10% polyacrylamide gel; the gel was dried and then imaged with E-Gel Imager. The frequency of the targeted integration was calculated by measuring the ratio of the cleaved to total product using ImageJ software. The percentage of apoptotic cells was determined using the Annexin-FITC apoptosis detection kit (Biyuntian, Beijing, China) according to the manufacturer’s instructions. The apoptotic cells were determined by flow cytometry (Beckman Coulter, Krefeld, Germany).

### Detection of exogenous gene insertion in bovine genome by PCR

The gene insertion was successively detected by 3′ junction PCR, 5′ junction PCR, and LR-PCR using EmeraldAmp® PCR Master Mix (TaKaRa). The 5′ junction PCR was conducted on the 3′ junction positive clones, whereas LR-PCR was performed on the 5′ junction positive clones. The three PCRs were performed using the primers 3cBF/R, 5cBF/R, and 5cBF/3cBR (Supplementary Table S1), respectively. The desired strands of junction PCR of the LR-PCR-positive clones were excised, purified using AxyPrep kit (Axygen, USA), and subcloned into pMD18-T vector (TaKaRa). The insert-containing colonies were sequenced and the precise location of the gene insertion was identified through blast searching of the *Bos taurus* genomic database of the National Center for Biotechnology Information (NCBI).

### SCNT

The SCNT procedures were performed as previously described^37^. In a typical procedure, *in vitro* matured oocytes were enucleated, and a single transgenic cell was injected into the perivitelline space. The couplets were fused using electrofusion. The reconstructed embryos were kept in synthetic oviductal fluid (SOF) containing 5 μg/ml cytochalasin B for 2 h. The fused embryos were then activated by 5 μM ionomycin and 2 mM 6-dimethylaminopurine. Following activation, the embryos were washed twice with mSOF and then cultured in 50 μL drops of mSOF medium covered with mineral oil at 38.5 °C, 100% humidity, and 5% CO_2_ in air. After 7 days of *in vitro* culture, two to three blastocysts were non-surgically transferred into the uterine horn of the synchronized recipient. Pregnancy was detected by rectal palpation and ultrasonography.

### rHSA detection and BLG expression in the targeted cows’ milk

The milk samples were collected as described previously^17^. In a typical procedure, the cloned female cows were induced to lactate by injections of 17 b-estradiol and progesterone. Five days after the intramuscular injection of dexamethasone, aseptic collection of milk was done by hand. The milk was defatted, and casein was removed by acid precipitation. The volume of isolated whey was also quantified. The resulting samples were then used for SDS-PAGE and Western blot analysis. For Western blot, the monoclonal anti-HSA antibody produced in mouse (Sigma, Missouri, USA) and the polyclonal anti-bovine β-BLG antibody produced in rabbit (Bioss, Beijing, China) were used as the primary antibodies (1:3000 and 1:500, respectively). The rHSA in the milk of the targeted cows was quantified using Human Serum Albumin ELISA Kit (KYM, Beijing, China) according to the manufacturer’s instructions. The absorbance values were measured at 450 nm using a 96-well plate reader Victor X5 (Perkin Elmer, Boston, MA, USA). A standard curve was constructed using the standard provided in the test kit, and the HSA concentration in the samples was calculated in the regression equations of the standard curve based on their OD values. Finally, the concentration of rHSA in milk was calculated by rHSA concentration in whey multiplied by the isolated ratio.

### Statistical analysis

Statistical significance for all data was determined using the SPSS 16.0 statistical software (IBM Corporation, Somers, NY, USA). Data were analyzed using one-way ANOVA and least-significant difference tests and were reported as mean ± SEM. *P* < 0.05 was considered statistically significant.

## Additional Information

**How to cite this article**: Luo, Y. *et al.* Generation of TALE nickase-mediated gene-targeted cows expressing human serum albumin in mammary glands. *Sci. Rep.* **6**, 20657; doi: 10.1038/srep20657 (2016).

## Supplementary Material

Supplementary Information

## Figures and Tables

**Table 1:** Preparation of gene-targeted cell clones.

Cell lines	Drug-resistant cell clones	3′-junction positive cell clones (%)	5′-junction positive cell clones (%)	LR-PCR positive cell clones (%)	Senescent cell clones*	Cell clones suitable for SCNT**
BFF10-20	317	27 (8.5)	21 (6.6)	18 (5.7)	5	13
BFF11-21	235	19 (8.1)	15 (6.4)	13 (5.5)	5	8
BFF07-23	283	15 (5.3)	12 (4.3)	9 (3.2)	4	5
Total	835	61 (7.3)	48 (5.7)	40 (4.8)	14	26

*No cell proliferation was observed in a week.

**The chromosome number of targeted cells was normal.

**Table 2:** Generation of gene-targeted cows by SCNT.

Donor cell lines	Cell clones	Reconstructed embryos	Embryos developed to blastocyst stage (%)	Recipients	Pregnancies at day 90 (%)	Calves at Birth	Calves survived for more than a month
BFF10-20	TH1020-6	198	45 (22.7)	16	5 (31.3)	2	1
	TH1020-23	215	50 (23.4)	17	3 (17.6)	3	2 (homozygous)
	TH1020-1	209	43 (20.6)	14	3 (21.4)	1	0
BFF11-21	TH1121-35	235	61 (26.0)	21	6 (28.6)	2	1
	TH1121-51	201	42 (20.9)	14	2 (14.3)	0	0
	TH1121-66	187	37 (19.8)	12	2 (16.7)	1	1
BFF07-23	TH0723-58	203	34 (16.7)	11	2 (18.2)	1	1
	TH0723-103	175	33 (18.9)	11	3 (27.3)	0	0
	TH0723-157	181	32 (17.7)	10	2 (20)	0	0
Total	—	1804	377 (20.9)	126	28 (22.2)	10	6
