# Supplementary material for: Generation of TALE nickase-mediated gene-targeted cows expressing human serum albumin in mammary glands
Source: Sci Rep. 2016 Feb 8;6:20657. doi: 10.1038/srep20657 (PMC4745098; doi:10.1038/srep20657)
Supplement: Supplementary Information [file srep20657-s1.pdf]

Generation of TALE nickase-mediated gene-targeted cows expressing human serum albumin in mammary glands

Yan Luo[ab], Yongsheng Wang[ab], Jun Liu[ab], Chenchen Cui[ab], Yongyan Wu[ab], Hui Lan[ab], Qi Chen[ab], Xu Liu[ab], Fusheng Quan[ab], Zekun Guo[ab] and Yong Zhang[ab]*

[a]College of Veterinary Medicine, Northwest A&F University, Yangling 712100, Shaanxi, China

[b]Key Laboratory of Animal Biotechnology of the Ministry of Agriculture, Northwest A&F University, Yangling 712100, Shaanxi, China

*Corresponding author: Yong Zhang, College of Veterinary Medicine, Northwest A&F University, Yangling 712100, Shaanxi, China

Tel.: (86) 029-87080085; Fax: (86) 029-87080085; E-mail: zhy1956@263.net

Supplementary methods and materials

Reporter system assay

The activity of TALENs was rapidly detected by red fluorescent protein–green fluorescent protein (RFP–GFP) reporter system [1]. Reporter vectors were reconstructed by insertion of target sequence of BLG into the HindIII/EcoRI between the RFP gene and an out-of-frame, functional GFP gene. TALENs-induced NHEJ at target sequence may restore GFP expression in the transfected cells. The target sequence was amplified from genomic DNA of non-transgenic cattle with primers E2SF and E2SR (Table S1). HK293 cells were seeded in 12-well plates at 0.8×10^5 before transfection. Upon reaching 80% to 90% confluence, the cell culture was changed with fresh Dulbecco's modified Eagle medium (DMEM, Gibco, New York, NY, USA) supplemented with 10% fetal bovine serum (FBS, HyClone, Logan, UT, USA) 6 h before transfection. Cells were cotransfected with 1 μg of TALEN expression vectors and 0.5 μg of corresponding reporter plasmid by FuGENE® HP Transfection Reagent (Roche, Mannheim, Germany) in accordance with the manufacturer's instructions. Cells transfected by reporter plasmids were used as negative controls. 36 h after transfection, RFP and GFP expression in transfected cells were observed under an inverted fluorescence microscope and the images were taken.

Primary bovine fetal fibroblasts culture

Bovine fetal fibroblasts (BFFs) were isolated from 45-day-old female fetus of Chinese Holstein dairy cows. The head and internal organs were discarded. The rest of fetus was minced extensively, seeded on 60 mm dishes wetted by Dulbecco's modified Eagle medium and Ham F12 (DMEM/F12, Gibco, New York, USA) plus 10% FBS (HyClone, Logan, UT, USA) and 100 IU/ml penicillin–streptomycin, and incubated at 37 ℃ for 4 h in an inverted manner. Up to 4 ml of fresh medium was then added. Upon reaching 80% to 90% confluence on the third or fourth day, the cells were digested by TE and passaged or cryopreserved.

Targeting vector construction

The targeting vector construction was conducted by the insertion of homologous arms surrounding customized TALENs' cleavage site, HSA coding gene with a signal peptide sequence of BLG at the upstream, BGHpolyA, and LoxP-EF1α-GFP-Puro-LoxP expression cassette into plasmid pMD18-T. Synthesized HSA coding gene was inserted into plasmid pMEL-b as previously described [2]. To reconstruct plasmid pgH, HSA coding gene BGHpolyA was amplified from pgH by PCR using primers hasF/R (Supplementary Table S1), and the purified PCR product was subcloned into pMD18-T to construct plasmid p18TH. The left and right arms were amplified from cattle genomic DNA by PCR with primers CarmL-F/R and CarmS-F/R (Table S1), respectively. The left arm was inserted into p18TH by endonuclease sites EcoRI and SnaBI to make plasmid pLH, and the right arm was inserted into pLH via SpeI and BsrGI to make pLRH. Finally, the Loxp-pEF1α-GFP-Puro-Loxp expression cassette

was digested from plasmid pBLG-puro, as described in reference [1], by SalI/SpeI and inserted into the AflII site by blunt end ligation to construct targeting vector pcB-HSA-puro. The vector structure is shown in Supplementary Fig. S1.

Selection of cell clones

The cells were seeded at 1×10^6 per 60 mm dish and cultured in DMEM/F12 plus 10% FBS. The cells were digested by TE (0.25% trypsin plus 0.05% EDTA), collected by centrifugation, washed with Opti-DMEM, collected by centrifugation, and resuspended by 400 μl electroporation buffer. The mixture of 20 μg TALENickase-encoding vectors and 10 μg pcB-HSA-puro was added into the cell suspension, and the mixture was transferred into 4 mm gap electroporation cuvette. After standing for 5 min, the cells were electroporated at 510 V with 1 pulse of 2 ms duration using the BTX Electro-cell manipulator. Finally, the cells were left to rest for 10 min, mixed with fresh cell culture medium, and seeded at 5×10^5 cells per 100 mm dish. After 2 days, the cell culture was changed with DMEM/F12 plus 10% FBS and 2 μg/ml puromycin. After selection for 6 days the clones were picked, transferred into 48-well plates and cultured with DMEM/F12 plus 10% FBS and 1μg/ml puromycin.

Off-target analysis

To test the off-target effect of the TALENs, Paired Target Finder [3] was used to predict

potential off-target sites in cow genome with the default criteria. Sequences of 9 potential off-target sites are listed in Table S2. We explored PCR to amplify the region around off-target sites with the primers provided in Table S2. The amplified products were sub-cloned into plasmid pMD-18T (TAKRA). 20 colonies for each sample were picked for extended culture and sequenced to confirm off-target cleavage. Gene mutation was analyzed by blast searching the *Bos Taurus* genomic database of the NCBI.

Southern blot

Genomic DNA was isolated from cloned cells or blood of cloned cows using Cell/Tissue Genome Isolation Kit (Tiangen Biotech, China) in accordance with the manufacturer's instruction. 20 μg genomic DNA was digested by restriction endonuclease Eco47III (NEB). The resultant product was separated on a 1% agarose gel at 25 V for 12 h. The DNA was denatured by gently rocking the gel in 10 volumes of denature buffer for 45 min, and neutralized for 45 min in 10 volumes of neutralization buffer. Finally, the separated DNA was transferred to a nylon membrane and detected using a DIG High Primer DNA Labeling and Detection Starter Kit II (Roche, Mannheim, Germany) referring to the instruction manual. For the preparation of 5′ probe external and HSA probe, DNA fragments of 624 bp and 538 bp were amplified using primers 5Pro-F/R and hPro-F/R, respectively (Table S1). The probes were created using the kit with the PCR products.

Protein characterization

Briefly, the milk from cloned cows and the natural milk spiked with pdHSA were defatted, and acid precipitation was done to remove the casein of the defatted milk. The resulting samples were loaded onto Pharmacia ABD column for HSA purification. Further purification was performed on Phenyl Sepharose HP (GE Healthcare). Finally, the HSA-containing fractions were concentrated, desalted, and freeze-dried. Purified rHSA and pdHSA were used for N-terminal amino acid sequence test by Edman degradation. Molecular mass was detected using Triple-TOF 5600^{+} (AB Sciex), and data was analyzed using Pro Mass 2.8. Circular dichroism (CD) spectroscopy in both the near (190 nm to 260 nm) and far (250 nm to 360 nm) UV spectra were performed on a JASCO 715 automatic spectropolarimeter with a scanning speed of 50 nm/min. Noise reduction was conducted by J7STDANL.

References

1. Cui, C. *et al.* Gene targeting by TALEN-induced homologous recombination in goats directs production of β-lactoglobulin-free, high-human lactoferrin milk. *Sci. Rep.* **5** (2015).
2. Luo, Y. *et al.* Chicken hypersensitive site-4 insulator increases human serum albumin expression in bovine mammary epithelial cells modified with phiC31

integrase. *Biotechnol Lett* **35**, 529-537 (2013).

3. Doyle, E. L. *et al.* TAL Effector-Nucleotide Targeter (TALE-NT) 2.0: tools for TAL effector design and target prediction. *Nucleic Acids Res* **40**, W117-W122 (2012).

Supplementary figure

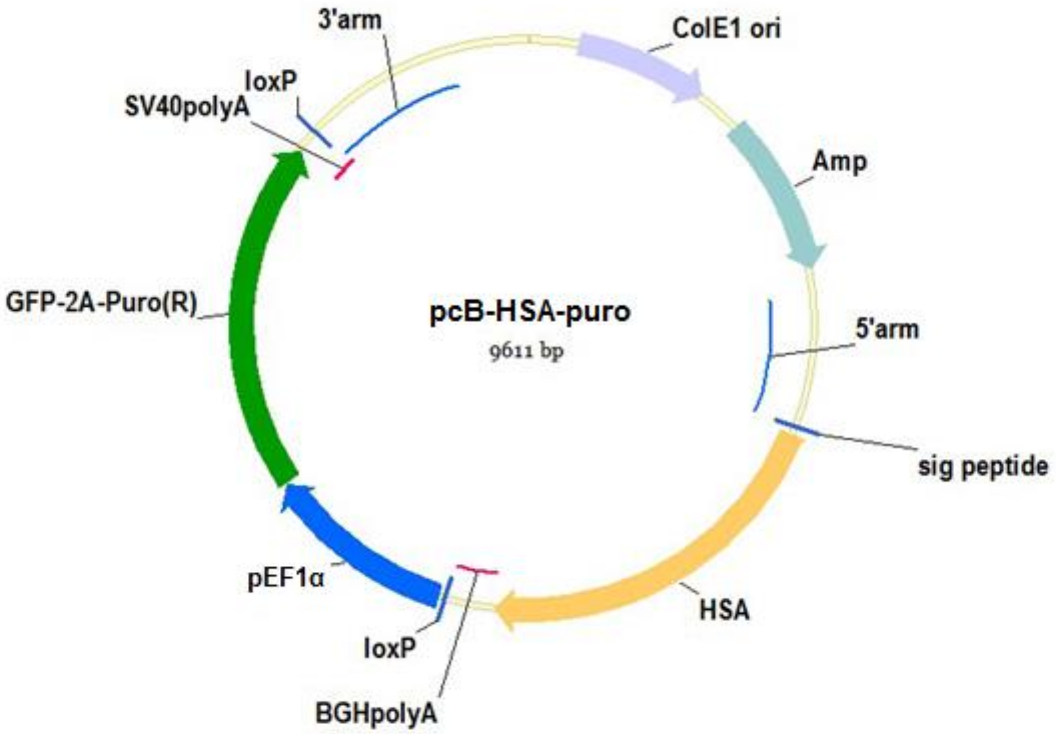

Fig.S1 Vector map of HSA gene targeting vector pcB-HSA-puro

Supplementary tables

Table S1 Primers used in vector construction and detection of cell clone

Primer name	Sequence (5′→3′)	Restriction enzyme	PCR product length
E2SF	AAGCTTGGGACTTGGTACTCCTTGGC	Hind III	180bp
E2SR	GAATTCCATATCCCTGGGGAGTGGGG	EcoR I	
Sebe-F	TGGTCTCCCCAAGATCCAAA	——	353bp
Sebe-R	CCATTTCTGCAGCAGGATCTC	——	
hasF*	TACGTAatgaagtgcctcctgcttgccctggccctcacttgtggcgcccaggccGATGCACACAAGAGTGAGGTTGCTCATC	SnaB I	2307bp
hasR	TGTACACGCCGACTAGTCTTAAGTCTTTCCGCCTCAGAAGCCATAGAG	BsrG I +Spe I +Afl II	
DarmF	ATTGTCACCCAGACCATGAAG	——	1571bp
DarmR	TTGGCTGCACTCACTGTCAC	——	
DinF	TGCAGAGCTCAGAAGCGTGAC	——	1829bp
DinR	CAATGATCTTCTTCTGAGCACACTC	——	
CarmL-F	GCTGAATTCATTGTCACCCAGACCATGAAG	EcoR I	723bp
CarmL-R	AATTACGTAACCTGGGGAGGGACCTTGAG	SnaB I	

CarmS-F	ACTAGTGGCGCGCCAGTGTATGTGGA GGAGCTGAAG	Spe Ⅰ	798bp
CarmS-R	TGTACATTGGCTGCACTCACTGTCAC	BsrG Ⅰ	
3cBF	TGTCCAAACTCATCAATGTATCTTA	——	1065bp
3cBR	CAATGATCTTCTTCTGAGCACACTC	——	
5cBF	TGCAGAGCTCAGAAGCGTGAC	——	968bp
5cBR	CAAATGGACACTGCTGAAGATACTG	——	
5Pro-F	TTCCCACCACTCCTGAGGTC	——	624bp
5Pro-R	CCTTCTTCCATTCGAGGCCAA	——	
hPro-F	AACACCCACTGATTTCTATGCTA	——	538bp
hPro-R	CATTGAAAACTGAGGAGTAACAC	——	

*Lower-case letters: sequence of signal peptide.

Table S2 Detection of the predicted off target sites in bovine genome

Genomic	Predicted off target sequence* (5′→3′)	Identity** (%)	PCR primer sequence (5′→3′)	Size of PCR products (bp)	Mutation
chr7	TGTCTGGAGCCTGATTTTgtg cctgtccgtgtgcctgtggtgAGTGTTA TTGGCCTGACT	52.8	gctgtgtgtgtatgttgcc caagggtcttctccaacacca	249	0
chr19	GGGCAAGGAGCCTGTGCTtg gctcacagcctaggctCCTGCCTGT TTTCCGCTG	50	agaagatgccctccggtct gaggtggggagtggtgtgtt	218	0
chr11	CACCTCACCCTTGGTGCTccc acaatactggataagAGTGTCAATC TCCTGAGA	50	agaaaccaacaggaccctgaa acagcataggggaagaatcacc	227	0
chr2	TGATTTCTATTGGTACTtagtta atctttccactctaagTTTGTTAACTA TATGTTA	45.7	tctttccccattttgcttcca cctttagtgcagtcctagacaa	231	0
chr11	GAGCAGAGAATTCAGCATag aattggggcaaaggtcaacaGGGTCC AAGTCTTAGTTG	44.4	agtgtcacgaacctgtgtcc gtggagggtggtatctacacg	198	0
chr10	TGGCAAAGTAATGTCTCTgct tttcaatatgctgtctacgttGGTCATAA CTTTCCTTCC	41.7	gcgctcagctttcttcacag taggctccaaaatcaccgca	207	0
chr11	TAAGGAAAAATTCTTACTcta	38.9	atccctttgtttccctggca	156	0

	gctactgcttatctaccagataTTTTATATGTATACGTAA		acaatatacataaggcctggtaaga		
chr11	AAACAAAAAGATCCTACTgtacagcacagggaacTATGTTCAATTTCCTCCA	38.9	acggagagtggttggagaga<br>actgctatacaggaaaatgactcag	201	0
chr2	TCACTACACAATGTTCCTcattttaaatatatttGGAACTGAGTTTCAGAAT	30.6	gcagtcagctttctcatgtcc<br>caggtgtgtgactgtggcaa	262	0

*Capitals: the left and right target sequences of TALENs, lower-case letters: sequences of spacer.

**The identity of TALE binding element sequence between the predicted off-target site and TALEN1/2.
